# Increasing Participation and Access to Economic Associations and Their Services

**DOI:** 10.1017/age.2020.21

**Published:** 2020-10-29

**Authors:** Elaine F. Frey, Jill L. Caviglia-Harris, Patrick Walsh

**Affiliations:** 1 Department of Economics, California State University, Long Beach, CA, USA; 2 California State University, Fullerton, CA, USA (current affiliation); 3 Department of Economics and Finance and Department of Environmental Studies, Salisbury University, Salisbury, MD, USA; 4 Manaaki Whenua – Landcare Research, Lincoln, New Zealand

**Keywords:** diversity, inclusion, professional development, economists, J71, A11

## Abstract

Discussions about increasing diversity in economics have ignored the role that associations play in the engagement of underrepresented economists. We continue work on diversity and inclusion in the Northeastern Agriculture and Resource Economics Association (NAREA) and other associations by analyzing membership and meeting attendance to promote diversity in economics. We estimate a vector error correction model (VECM) to identify the determinants of membership and meeting attendance and use member survey data to model membership and meeting attendance behavior. We find inequalities across gender, income, and professional status. Recommendations include locating meetings in accessible cities, increasing networking opportunities, and providing more services supporting underrepresented groups.

## Introduction

Economics is not a gender or racially diverse field (Bayer and Rouse 2016; Collins 2000; McDowell, Singell, and Ziliak 1999). And even though this lack of diversity has been a point of discussion for at least four decades, the field continues to be noted as toxic for women and one in which minorities are largely underrepresented (AEA 2019; Wu 2017).^1^ These inequalities are observed as early as in undergraduate degree programs, where the percentage of women is lower than in many other predominately male fields (Bansak and Starr 2010) and continues throughout graduate programs and careers in the form of less representation in academic ranks and the publications needed for career advancement (Bayer and Rouse 2016; West et al. 2013).^2^

Although initiatives to recruit more women undergraduate majors have been under-taken, and programs to support the early-career pipeline through mentoring programs have recently made headway (Bayer and Rouse 2016; Rouse 2009), progress has not been rapid (Kahn 1995). This was made all too clear when Wu (2017) quantified the degrading language used to identify women economists on the now notorious Economics Job Market Rumors website. A few associations are now working to improve the climate of the profession (AEA 2018a; 2018b). In particular, it is the work of the Northeastern Agricultural and Resource Economics Association (NAREA), and the call by Sarah Jacobson (2018) to do more to improve upon diversity and inclusion, that we contribute to by analyzing organizational and survey data on membership and association benefits in order to identify concrete steps that associations can take to increase diversity in economic associations and association leadership positions.

Professional association meetings provide important networking opportunities (Garfield 1991), can have a positive impact on citation rates, and are an essential part of the scientific process, as they encourage peer input into the most recent advances of the field (Di Vaio, Waldenstrom, and Weisdorf 2012; Taylor, Fender, and Burke 2006). While a significant number of PhD economists become members of at least one professional economic association over the course of their careers, the benefits of these associations, and the meetings they support, tend to favor those who need these benefits least. For example, graduate students and postdocs face a greater number of obstacles compared to academic faculty when it comes to having a paper accepted to a meeting and obtaining the funding needed to attend (Garfield 1991). And women are often underrepresented as keynote speakers (Lundberg and Stearns 2019), less likely to engage in question-and-answer sessions (Gunther, Grosse, and Klasen 2017), and more likely to experience harassment (Shinall 2018). Furthermore, the “childcare-conference conundrum” affects mothers more than fathers (Calisi 2018).^3^ While discussions about increasing diversity in economics have noted the role of mentoring, the pipeline, and undergraduate exposure, the role that associations and meetings can play in advancing the careers of women and minority groups has been largely overlooked in the literature.

One overlooked way of supporting the careers of women and minority economists is by encouraging their participation in associations and their annual meetings. The limited research on economic associations focuses on identifying drivers of membership and meeting attendance. Evidence suggests that membership numbers are linked to the number of PhDs and PhD programs (Kilmer 2004), the annual meeting date and location, and journal access. Meeting dates that are more accommodating to academic schedules and meeting locations that are easily accessible are relatively less costly (Siegfried and Nelson 1979) and often translate into more meeting attendees and association members in any given year (Siegfried 2002; Broder, Bergstrom, and Kriesel 1994). Finally, access to and quality of the association journal has historically been an important membership benefit (Siegfried 2002). Our study updates the outdated literature in this area, uses a more sophisticated empirical strategy, and is more comprehensive because we also use member survey data. We provide insights on issues not considered in previous research, such as how gender, income status, and professional status affect meeting attendance. Findings from our paper suggest that although there is no gender gap in membership, there is a gender gap in meeting attendance. Women do seem to face additional barriers to attending meetings and accessing their associated career benefits. Similarly, lower income status is also a barrier to attending meetings.

This paper identifies the determinants of membership and meeting attendance on two different scales to identify policies that can increase participation and diversity in the profession and to highlight different policy prescriptions for large and small associations (like NAREA). The term diversity can refer to gender identity, age, race, ethnicity, nationality or national origin, sexual orientation, religion, disability, health condition, physical appearance, marital status, parental status, socioeconomic status, professional status, or personal connections. Due to data availability, we are only able to focus on socioeconomic status, gender, and professional status and to refer to these aspects as diversity in the paper, although we readily admit that other elements of diversity are equally important – if not more – to study at the present in time.

The remainder of this paper proceeds as follows: in the next section, “Data and Descriptive Statistics,” we describe the membership and survey data that we have collected from external sources and our own survey. Then, in the following section, “Empirical Methodology and Results,” we use two empirical strategies to identify policies for increasing participation in economics associations and annual meetings. Because our data are non-stationary, we use a vector error correction model (VECM) to analyze time-series data on membership and meeting attendance of three associations over time and to identify the impact of policy on membership and attendance. In addition, we use a Poisson model to estimate individual membership and attendance while controlling for socioeconomic characteristics of members. We consider the role of socioeconomic status, gender, and professional status in the determinants of these models and provide practical recommendations on how NAREA and different types of associations can sustain or increase the diversity of their membership and participation in their meetings. In the “Conclusions” section, we offer with suggestions for how our findings can be used to increase diversity in economics.

## Data and Descriptive Statistics

Two sources of data are used to investigate the determinants of association membership and meeting attendance. They are a 40-year panel from three large economics associations and survey data from 2,931 members of four associations. We group economic associations into four categories: national, regional, field, and regional-field. The American Economic Association (AEA), founded in 1885, is a national association in the United States (Bernstein 2008). The four primary regional associations include the Eastern Economic Association (EEA), Midwest Economics Association (MEA), Southern Economic Association (SEA), and Western Economic Association International (WEAI).^4^ There are other regional associations in this category, which target smaller geographic areas but remain open to all subdisciplines, such as the New York State Economics Association. Field associations target a subdiscipline within economics and have no regional boundaries, such as the Agricultural and Applied Economics Association and Urban Economics Association. Finally, regional-field associations target specific subdisciplines within a regional boundary, such as NAREA and Midwestern Law and Economics Association.

### Association Time-Series Data

Economic association data on membership, dues, meeting attendance, meeting registration fees, and meeting locations for the 40-year time period after 1975 (or the first year available) were obtained from Siegfried (2002) (which has data on AEA membership history), association records, staff, and journals for the AEA, SEA, and WEAI.^5^ These data suggest that average AEA membership increased from over 19,000 in the 1970s to an average of 21,109 between 1986 and 1995. Then between 2006 and 2015 it declined to 17,945 (see Table 1). Average SEA membership fell from a total of 1,733 between 1975 and 1985 to approximately 1,400 between 1986 and 1995, fell again in the decade that followed, but then increased to 1,536 between 2006 and 2015. Average WEAI membership peaked at 2,053 in the 1986 to 1995 time period and fell in each time period since. Concurrently, average attendance at the annual meetings increased by 97, 27, and 74 percent, respectively, for the AEA, SEA, and WEAI between 1975 (or the earliest year for which we have data) and 2015. Membership dues increased significantly for each association over the period 1975–2015, with SEA dues increasing almost 800 percent, although the AEA dropped its dues from $98 to $40 in 2013. At the same time, real conference registration fees also increased for all associations over this period at a higher rate than membership fees, with the average SEA registration fees increasing by more than 2000 percent.^6^ Table 1 also contains summary statistics on dummy variables for meeting locations in the largest cities within the regions and regular meeting rotations, since these cities are expected to attract more meeting attendees due to the ease of travel and relatively lower travel costs (Siegfried 2002; Siegfried and Nelson 1979). These cities include New York; Washington, DC; Chicago; and Philadelphia for AEA; New Orleans and Washington, DC for SEA; and Los Angeles and San Francisco for WEAI. The AEA never held a meeting in Los Angeles, while the SEA and WEAI (domestic) never held meetings in the other cities outside of their locational centers.

A Dickey-Fuller (DF) test suggests that many of the variables included in our models of AEA, SEA, and WEAI membership and attendance are non-stationary (see Table 2) and therefore that including them in the time series analysis would produce unreliable and spurious results (Johansen 1991; Engle and Granger 1987). We do, however, find the differenced variables to be stationary and therefore stationarize the time series by estimating the year-to-year differences of the independent variables (see Table 2).

### Member Cross-Sectional Survey Data

Our second set of data includes survey responses from 2,931 participants from four economics associations, including one national, regional, field, and regional-field association (NAREA).^7^ In each case, members were emailed an invitation to participate in an online survey. Two follow-up emails were sent to each group, resulting in a response rate ranging from 16 to 44 percent (see Table 3). Response rates are slightly lower if we only include completed surveys for variables of interest.^8^ The survey instrument was identical across associations, except for a few tailored questions added by the associations themselves (not reported here). The first part of the survey gathered information on respondent membership benefits, ranking of the reported benefits, information on membership status, and attendance at the association’s annual meeting over time. The second part of the survey gathered demographic information, including employment status, type of employer, field of expertise, educational attainment, gender, and income.

Table 4 contains descriptive statistics for several key survey questions. We find that between 28 and 68 percent of the survey respondents indicate that their association provides benefits that other economics associations do not. On average, respondents in the national association hold their membership longer (10–20 years) than the other associations (2–5 years). Approximately 44–64 percent of association members pay membership fees out-of-pocket. Respondents of the national and field associations are on average members of three associations, compared to four associations for the others. Regional associations appear to attract higher proportions of respondents employed by universities, with 89 percent, compared to 66–78 percent in other associations. The percentage of respondents that hold a doctorate is highest for the regional association (96 percent). The percent of female respondents, which is likely representative of female participation in the association, is lowest for the national association (20 percent) and increases as the association size decreases and specialization of the association increases. Approximately 30 percent of NAREA (regional-field association) respondents are female. For reference, the share of female economics PhDs in 2016 was 31 percent, a figure that has remained fairly constant in the U.S. over the last 20 years (Lundberg 2018). The average household income is between $120,000 and $159,000 for respondents of the national association and lower for the respondents of other associations.^9^

**Figure 1.**
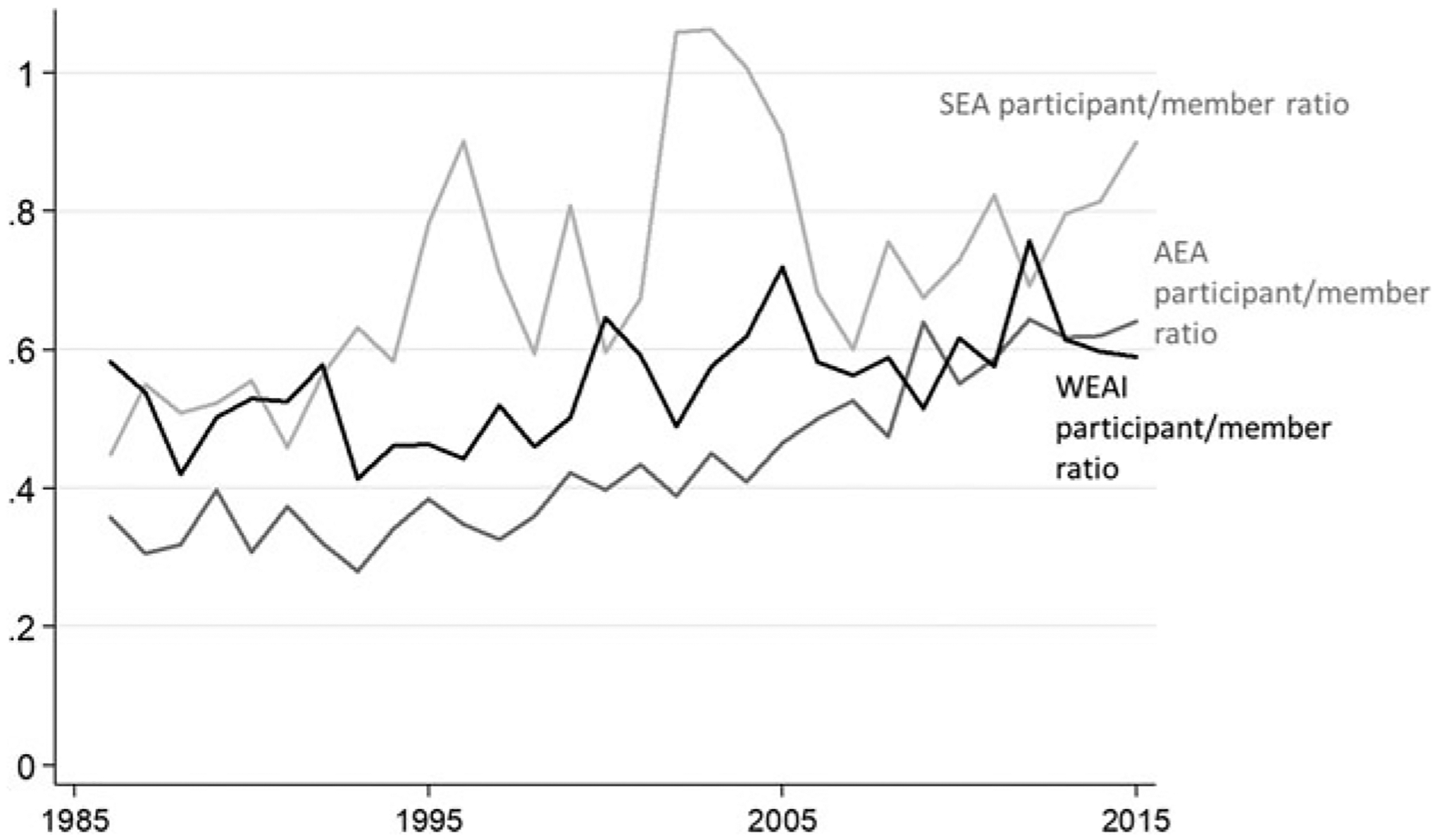
Ratio of Meeting Attendees to Association Members from 1986 to 2015 for AEA, SEA, and WEAI

The top-ranked benefits reported for associations include access to the association journal, the annual meeting, job market opportunities, staying professionally current, and networking (see Table 5). With the exception of the national association, up to 64 percent or more respondents note that networking and the annual meeting are the most important benefits of the association (combined). For NAREA, networking is ranked as the most important benefit of membership. Respondents prefer to maintain membership with more than one economic association, and they are neutral in the belief that cost is an important determinant of membership (see Table 6). Given that members receive different benefits from different types of associations, it’s not surprising that most respondents prefer to maintain more than one membership. Members of all associations believe that location is a marginally important determinant in the decision to attend the annual meeting and prefer to attend meetings that are close to airports. Respondents are on average indifferent about whether they maintain membership only when they attend the annual meeting; although our estimations in the next section suggest that attendance at the annual meeting is becoming a more important influence on maintaining memberships. Our membership data suggest that the ratio of meeting participants to association members is increasing over time (see Figure 1).

## Empirical Methodology and Results

We use two empirical strategies to identify policies to increase participation in economics associations and annual meetings. First, because our data are non-stationary, we (1) use a vector error correction model (VECM) to analyze time-series data on membership and meeting attendance of three associations over time, and (2) identify the impact of various policies imposed by associations on membership and attendance. Second, we use a Poisson model to estimate individual membership and attendance while controlling for socioeconomic characteristics of members.

### Association Time-Series Models and Results

We first estimate the factors that drive membership and meeting attendance over time. We assume that the time-series relationship for membership in time *t* (*M*_*t*_) is given by equation (1), where total association membership is a function of membership dues (*P*_*t*_), lagged membership (*M*_*t-1*_), meeting attendance (*A*_*t*_), the number of new economics PhDs earned in the U.S. (*PhD*_*t*_), national gross domestic product (*GDP*_*t*_), dummies for association policy changes (*Policy*_*t*_), and locational dummies for the annual meeting (*X*_*t*_).


(1) $$M_{t}=f\left(P_{t},M_{t-1},A_{t},PhD_{t},GDP_{t},{\text{Policy}}_{t},X_{t}\right)$$

Similarly, the relationship for meeting attendance (total members to attend the annual meeting) is given by equation (2), where the number of attendees is a function of meeting registration fees (F_t_), lagged meeting attendance (A_t-1_), the number of new economics PhDs earned, national gross domestic product, dummies for association policy changes, and location dummies for the annual meeting.


(2) $$A_{t}=f\left(F_{t},A_{t-1},PhD_{t},GDP_{t},{\text{Policy}}_{t},X_{t}\right)$$

We estimate membership and meeting attendance models for the time series we have for each association (1970–2015 for AEA, 1975–2015 for SEA, and 1982–2015 for WEAI). The general VECM model takes the general form

(3) $$\Delta x_{t}=\beta_{1}^{\left(m\right)}\Delta x_{t-1}+\beta_{2}^{\left(m\right)}\Delta x_{t-2}+\ldots +\beta_{k-1}^{\left(m\right)}\Delta x_{t-k+1}+\delta x_{t-m}+\Phi D_{t}+\varepsilon_{t},$$

where *m* is an integer between 1 and *k* that defines the lag placement of the VECM term, k is the lag length, and the deltas represent the differenced independent and dependent variables (to make our data stationary). We identify the number of lags to be included using the Johansen integration test (Johansen 1991) and estimate the model with these lags (see Table 7). Tests for cointegration identify such evidence in the estimations of membership and meeting attendance for all three associations (see Table 8). Our final models are chosen based on the results from the lag selection (see Table 7) and Johansen tests for cointegration (see Table 8). The lags and ranks are also noted in the VECM estimations presented in Table 9.

We include at least one policy variable in each of our estimations.^10^ For AEA we identified two policy changes over this time period. First, in 2000 the association began to require at least one paper author be a member of AEA or one of the other affiliated associations (policy 1), and second, in 2009 AEA began to require that users of the online hotel reservation system for the conference were members (policy 2). For SEA we identified one policy change in the registration fee structure: in 2004 the meeting registration fee was higher for non-members. We identified one policy change for WEAI. In 1994 WEA added “International” to its name and began to hold (in addition to the annual meeting) biennial meetings in the Pacific Rim. It is likely that other policy changes, particularly in the early years of our dataset, also took place. If so, these policy changes are not in the institutional memory of these associations as far as we can discern. AEA has never charged different registration fees for members and non-members and WEAI has always charged a higher rate for non-members.

We discuss each association’s short-run effects (see Table 9) and begin with the estimation of AEA membership and meeting attendance. We focus on the discussion of the policies noted above and the location decision.^11^ The short-run results include differenced lags of the controls (GDP, lagged attendance, prices, conference registrants, and new PhDs) and policy and meeting location dummy variables (long-run results only include differences and thus no policy variables).^12^ The results suggest that locating the annual meeting in New York increases attendance by 1,572 and locating the annual meeting in Washington, DC, increases attendance by 1,800. However, locating the annual meeting in these locations does not translate into increases in membership. The policy to require at least one paper author be a member of AEA (policy 1) is correlated with 1,894 fewer conference registrants and 2,196 fewer members. The requirement that attendees must be members to register in the meeting hotels (policy 2) is correlated with an increase of 2,164 conference registrants, but this policy was not correlated with an increase in membership.

We continue with the estimation of SEA membership and meeting attendance, where the short-run results suggest that locating the annual meeting in New Orleans increases attendance by 133 and locating the annual meeting in Washington, DC, increases attendance by 175. Locating the meeting in Washington, DC, also translates into an increase of approximately 149 new members, but there is no impact on membership when the annual meeting is held in New Orleans. These positive and significant Washington, DC, conference attendance results for both SEA and AEA make sense, since the city has the largest concentration of economists in the country.^13^

We conclude with the estimation of WEAI membership and meeting attendance. According to our results, the policy initiated in 1994 to become WEA International had negative impacts on meeting attendance. According to our results, this decision decreased attendance at the annual meeting by 268. This might be because members treat the domestic and international meetings as substitutes, resulting in a decline in meeting attendance. Finally, the results suggest that locating the annual meeting in San Francisco increased membership by 167 members.

In sum, these estimations suggest that the findings from some previous less-sophisticated models (Siegfried 2002; Siegfried and Nelson 1979) continue to hold: meeting locations in the largest cities attract more meeting participants. We also find that a portion of these meeting attendees are translated into association members. With these more general findings in mind, we advance our understanding of the type of members that are most impacted by these decisions with individual level data on perceived benefits of association meetings, meeting attendance, and association membership choices.

### Association Survey Model and Results

While the estimations above can point to policies that can increase membership and annual meeting attendance, these policies do not address diversity, since they are based on association and not individual data. To explore policies that target different socioeconomic groups, we estimate membership and attendance with survey data and the Poisson model given by:

(4) $$Q_{i}=\beta_{0}+\beta_{1}P_{i}+\beta_{2}I_{i}+\beta_{3}S_{i}+\varepsilon_{i}$$

where individual *i*’s count of memberships or meetings attended each year (*Q*_*i*_) is a function of price (*P*_*i*_) that is proxied by an indicator of payment out of pocket, household income (*I*_*i*_), and a series of socioeconomic characteristics (*S*_*i*_), including the number of years employed, gender, holding a PhD, university employment, and years of membership. The stochastic disturbance term (*ε*_*i*_) represents the net impact of all unobservable factors that influence the dependent variable.^14^

Results in Table 10 suggest that members of the regional and regional-field associations (NAREA) hold more association memberships than those from the national association, the comparison unit. This is an expected result, since regional-field members are likely to be regional or national members, but national members are not necessarily regional-field members. But NAREA members and field association members do not attend more meetings, compared to those from the national association. Regional association members attend more meetings, compared to national members. This makes intuitive sense, since a higher percentage of regional members find presenting a paper to be the most valuable benefit of meetings, compared to all other types of members (see Table 5). Field association members are not distinguished from national association members in memberships or meeting attendance. The number of years a respondent has been a member of their association is also positively associated with the number of memberships held, but not with meetings attended. As expected, in Model 1 and 2, attending more meetings has a positive impact on the number of memberships. Models 2 and 4 are included to identify if there is a tenure effect. We expect that because teaching and research incentives can differ before and after a faculty member obtains tenure, these different incentives may impact association membership and attendance at meetings. In Model 2, we do see this effect, since the dummy variable for those with less than six years employment is positive and statistically significant.

Duration and type of employment affects the number of memberships and meetings attended. University employees are members of more associations and attend more meetings than others, all else being equal. However, those who have worked at a university for more than six years, a proxy for the post-tenure period, attend fewer meetings compared to respondents with less than six years at a university (Models 3 and 4). The number of years employed is negative and significantly related to the number of meetings, but has a positive impact on the number of memberships in Model 1. We also identify what we term a tenure effect: faculty who have been employed for more than six years are less likely to attend meetings (Model 4). Together, these results suggest that early-career scholars attend conferences and present their research more often, during what is likely the pre-tenure years and then reduce the number of meetings per year as careers advance, but memberships are still maintained.

There are several interesting insights that can be drawn across demographic characteristics. First, household income does not affect the number of memberships economists hold, but it does affect how many meetings per year economists attend. Respondents who self-pay tend to hold more memberships but attend fewer meetings. Given the large expense of travel compared to association membership, it makes intuitive sense that lower household incomes and those who self-pay attend fewer meetings per year. Respondents that hold a PhD do not maintain more memberships, but they do attend more meetings. Another interesting result is that male economists do not appear to hold more memberships than female economists, but males do attend more meetings per year compared to females, suggesting that females face greater barriers for meeting attendance. This implies that there is no gender gap in terms of association memberships, which have little impact on career trajectory (since anyone can pay a membership fee), but a gender gap does exist for meeting attendance, which prevents women from accessing important opportunities for professional development.

## Conclusions

This study analyzes time-series association data and cross-sectional survey data from a large sample of economic association members to draw conclusions on how NAREA and other associations can increase the diversity of association membership and meeting attendance. Our empirical results allow us to identify several barriers to meeting access. First, income status can be a barrier to meeting attendance. Because cost of travel is closely linked to meeting location and opportunity cost, this is one policy area under the direct control of association leadership. Similar to previous work, we find that meeting locations that are easily accessible and are relatively less costly (Siegfried and Nelson 1979) translate into more meeting attendees and association members in any given year (Siegfried 2002; Broder, Bergstrom, and Kriesel 1994). Locating meetings in hard-to-reach locations disadvantages parents and those with lower levels of research funding. This points to implications about equitable access to meetings for economists across a broad range of institutions, with those from less prestigious institutions likely having less institutional support for travel. Results from member surveys confirm that those who pay out-of-pocket attend fewer meetings per year, which is more likely to be economists from less prestigious institutions.

Although AEA has taken measures to reduce membership fees for lower income levels, our results suggest that income is typically not a barrier to becoming a member. Survey data suggest that members do not place much importance on membership dues or meeting registration fees. Yet graduate students or low-income individuals may be more sensitive to membership fees, because they are less likely to have institutional support for travel. Subsidies or grants for student participants, new members, and low-income individuals could help provide better access to association services and help increase the diversity of members.

Our survey data also reveal heterogeneity across membership characteristics and perceived benefits: while a majority of association members are academic economists, the national association members tend to have higher incomes and participate in fewer annual meetings and associations. Furthermore, we find that the importance of networking increases as association size decreases. This points to the rationale for economists to maintain multiple memberships, since each association provides different professional development opportunities. NAREA and associations that are relatively small and focused on a region or field would therefore benefit from events that build on this strength, such as cocktail hours, meet-and-greet opportunities, mentoring programs, meeting field trips, and opportunities to participate in governance; networking services should specifically target those who are underrepresented in the profession. Associations can also build upon technological advances to supplement in-person networking opportunities. For example, social media, virtual meeting experiences, or mentoring experiences not directly tied to meeting attendance may be more equitable for those facing barriers to attending meetings and are likely to become more common and more advanced in a post-pandemic world. Of course, the global pandemic may pose different barriers to women and people of color, which needs to be carefully considered. Associations that are large and display other core competencies, such as job market activities and high-quality journals, should improve upon and maintain these services. Reducing bias in job market activities and publishing should be a priority for these associations.

Finally, we find important differences across demographic characteristics. We provide evidence that inequalities in meeting attendance do exist for women, lower-income individuals, and non-academic economists. We believe that underrepresented groups not directly analyzed in this paper face even greater barriers. Our time-series analysis of meeting attendance and membership show that association policy changes can influence membership. Furthermore, because our estimations also suggest that female economists attend fewer meetings than males but do not hold fewer memberships, we believe this points to inequalities and additional barriers women may face to attend meetings. To change this, associations should increase their support of women and underrepresented minorities by providing a more inclusive environment at meetings, providing meeting childcare services, providing more professional development opportunities targeting these members, and exploring other policy options with the groups of interest. NAREA has taken a leadership role in these endeavors by creating a committee on diversity and inclusion and starting several new initiatives to attract and elevate diverse members. We suspect these findings can help target their next steps.

## Figures and Tables

**Table 1.** Descriptive Statistics of Economic Association Data from 1971 to 2015

	AEA				SEA				WEAI			
	1975–1985	1986–1995	1996–2005	2006–2015	1975–1985	1986–1995	1996–2005	2006–2015	1982–1985	1986–1995	1996–2005	2006–2015
Members (number)	19151.5	21109.1	19717.6	17945.2	1733.4	1399.7	1102.0	1536.1	1908.8	2052.9	1930.0	1780.5
	(993.8)	(688.7)	(1194.0)	(1152.1)	(87.05)	(141.0)	(90.92)	(167.7)	(296.4)	(337.8)	(177.1)	(119.2)
Membership Dues ($2015)	13.61	35.79	65.84	77.81	7.349	24.21	42.60	65.87	21.75	27.95	46.90	63.18
	(5.574)	(8.898)	(9.753)	(24.75)	(3.742)	(6.948)	(6.799)	(9.539)	(4.966)	(5.840)	(5.014)	(5.952)
Conference Attendees (number)	4816.7	7139.6	7843.2	10434.2	913.3	773.8	908.0	1158.3	712.5	1038.8	1168.1	1239.5
	(1386.4)	(836.4)	(575.6)	(1583.6)	(184.8)	(69.13)	(170.6)	(259.9)	(74.64)	(194.1)	(171.2)	(245.3)
Conference Registration Fee ($2015)	6.189	17.41	39.10	63.10	7.971	35.30	62.64	168.8	32.28	51.99	93.21	159.3
	(3.129)	(4.870)	(7.615)	(6.859)	(5.049)	(12.52)	(9.283)	(41.31)	(6.863)	(8.336)	(19.78)	(21.12)
Conference in New York (dummy)	0.182	0.200	0.100	0.000	0.000	0.000	0.000	0.000	0.000	0.000	0.000	0.000
	(0.405)	(0.422)	(0.316)	(0.000)	(0.000)	(0.000)	(0.000)	(0.000)	(0.000)	(0.000)	(0.000)	(0.000)
Conference in Washington DC (dummy)	0.0909	0.200	0.100	0.000	0.273	0.200	0.300	0.200	0.000	0.000	0.000	0.000
	(0.302)	(0.422)	(0.316)	(0.000)	(0.467)	(0.422)	(0.483)	(0.422)	(0.000)	(0.000)	(0.000)	(0.000)
Conference in Chicago (dummy)	0.0909	0.100	0.100	0.200	0.000	0.000	0.000	0.000	0.000	0.000	0.000	0.000
	(0.302)	(0.316)	(0.316)	(0.422)	(0.000)	(0.000)	(0.000)	(0.000)	(0.000)	(0.000)	(0.000)	(0.000)
Conference in Philadelphia (dummy)	0.000	0.000	0.100	0.100	0.000	0.000	0.000	0.000	0.000	0.000	0.000	0.000
	(0.000)	(0.000)	(0.316)	(0.316)	(0.000)	(0.000)	(0.000)	(0.000)	(0.000)	(0.000)	(0.000)	(0.000)
Conference in New Orleans (dummy)	0.000	0.200	0.200	0.100	0.273	0.400	0.300	0.300	0.000	0.000	0.000	0.000
	(0.000)	(0.422)	(0.422)	(0.316)	(0.467)	(0.516)	(0.483)	(0.483)	(0.000)	(0.000)	(0.000)	(0.000)
Conference in Los Angeles (dummy)	0.000	0.000	0.000	0.000	0.000	0.000	0.000	0.000	0.250	0.100	0.000	0.000
	(0.000)	(0.000)	(0.000)	(0.000)	(0.000)	(0.000)	(0.000)	(0.000)	(0.500)	(0.316)	(0.000)	(0.000)
Conference in San Francisco (dummy)	0.182	0.000	0.100	0.100	0.000	0.000	0.000	0.000	0.000	0.200	0.300	0.100
	(0.405)	(0.000)	(0.316)	(0.316)	(0.000)	(0.000)	(0.000)	(0.000)	(0.000)	(0.422)	(0.483)	(0.316)

Notes: Standard deviations in parentheses.

**Table 2.** Dickey-Fuller Unit Root Tests

	AEA (1970–2015)				SEA (1975–2015)				WEAI (1982–2015)			
	Original Value		Differenced Value		Original Value		Differenced Value		Original Value		Differenced Value	
	Statistic	p-value	Statistic	p-value	Statistic	p-value	Statistic	p-value	Statistic	p-value	Statistic	p-value
Members (number)	−1.536	0.8163	−6.278***	0.000	−0.250	0.9906	−7.143***	0.000	−1.872	0.6693	−4.530***	0.001
Membership Dues ($2015)	−1.105	0.9282	−6.553***	0.000	−2.277	0.4467	−7.700***	0.000	−2.445	0.356	−5.218***	0.000
Registration Fee ($2015)	−2.468	0.3439	−7.962***	0.000	−0.759	0.9690	−6.781***	0.000	−1.435	0.8504	−4.713***	0.001
Registrants (number at annual meeting)	−4.507***	0.0015	−11.359***	0.000	−2.136	0.5259	−6.985***	0.000	−5.942***	0.0000	−14.074***	0.000
PhDs (number of new)	−3.955***	0.0102	−8.144***	0.000	−3.440**	0.0462	−7.658***	0.000	−3.32*	0.0627	−6.909***	0.000
Gross Domestic Product, GDP (real)	−1.955	0.6257	−4.424 ***	0.000	−1.790	0.7094	−4.066***	0.007	−1.46	0.8424	−3.579**	0.032

Notes: The null hypothesis is that the variable is non-stationary;

* significance at the 10%;

** significance at 5%;

*** and significance at 1%.

**Table 3.** Response Rates of Economic Association Surveys

	Population Emailed	Number of Respondents	Response Rate
National Association, Round 1	30,364	2,104	6.93%
National Association, Round 2	5,868	1,039	17.71%
Regional Association	2,318	479	20.66%
Field Association	1,800	419	23.28%
Regional-Field Association (NAREA)	284	124	43.66%

Notes: The response rate for the national association is estimated to be approximately 16%. The first round of the survey was administered to an old, but public, listing of members. The second round of the survey was sent to a subset of current association members (respondents were not permitted to complete the survey twice). The combined rounds divided by the current membership is approximately 16%.

**Table 4.** Descriptive Statistics from Economic Association Surveys

Variable Name	Definition	National Association (n = 2,244)	Regional Association (n = 311)	Field Association (n = 284)	Regional-Field Association (NAREA) (n = 92)
Membership Years	Number of years of membership in organization; 1 = less than one year, 2 = 1 year, 3 = 2–5 years, 4 = 5–10 years, 5 = 10–20 years, 6 = 20–30 years, 7 = more than 30 years	4.844	3.653	3.680	3.717
		(1.570)	(1.736)	(1.545)	(1.731)
Membership Benefits	Indicator of whether organization provides unique membership benefits; 1 = yes, 0 = no	0.676	0.284	0.467	0.584
		(0.468)	(0.452)	(0.500)	(0.496)
Self-Pay	Indicator of self (out of pocket) payment for organization membership; 1 = yes, 0 = no	0.640	0.441	0.542	0.609
		(0.480)	(0.497)	(0.499)	(0.491)
Associations	Number of professional associations (in addition to organization) that respondents have membership in	3.161	3.797	3.257	3.565
		(1.518)	(1.475)	(1.389)	(1.536)
Meetings	Number of professional economics meetings attended in an average year	2.787	3.232	3.042	3.033
		(1.524)	(1.233)	(1.226)	(1.330)
University	Employment at a university; 1 = yes, 0 = no	0.662	0.894	0.778	0.739
		(0.473)	(0.308)	(0.416)	(0.442)
PhD	Holds a PhD degree; 1 = yes, 0 = no	0.868	0.961	0.933	0.902
		(0.339)	(0.193)	(0.250)	(0.299)
Female	Female; 1 = yes, 0 = no	0.204	0.293	0.289	0.304
		(0.403)	(0.456)	(0.454)	(0.463)
Income	Household income (before taxes); 1 = less than 40,000, 2 = 40,000–79,000, 3 = 80,000–119,000, 4 = 120,000–159,000, 5 = 169,000–199,000, 6 = 200,000 and over	4.284	3.920	4.144	4.054
		(1.498)	(1.395)	(1.510)	(1.261)
Years Employed	Number of years at current position; 1 = less than one year, 2 = 1–3 years, 3 = 3–5 years, 4 = 5–10 years, 5 = 10–20 years, 6 = more than 20 years	4.400	3.617	3.715	3.880
		(1.458)	(1.620)	(1.536)	(1.676)

Note: Standard deviations in parentheses.

**Table 5.** Highest Ranked Association Benefit by Association Type (Survey Data)

	National Association	Regional Association	Field Association	Regional-Field Association (NAREA)
	(n = 2,244)	(n = 311)	(n = 284)	(n = 92)
Access to the association journal	50.58	17.50	24.82	22.22
Annual meeting	19.60	45.96	33.75	20.83
Networking opportunities	14.26	28.47	39.24	43.40
Ability to be active in the profession	20.51	14.04	19.35	19.57
Job market opportunities	23.27	0.00	3.571	0.00
Governance	3.947	8.333	0.00	0.00

(top ranked benefit; percent of respondents to note benefits)

**Table 6.** Association Preferences by Association Type (Survey Data)

	National Association	Regional Association	Field Association	Regional-Field Association (NAREA)
	(n = 2,244)	(n = 311)	(n = 284)	(n = 92)
Prefer to be a member of more than one association	3.697	3.952	3.838	3.902
	(1.006)	(0.847)	(0.891)	(0.890)
Cost is a factor in membership decisions	3.212	3.416	3.327	3.370
	(1.172)	(1.102)	(1.071)	(1.076)
Membership in a national association is more important than in a field association	2.834	2.500	2.366	2.391
	(1.067)	(1.020)	(1.005)	(1.037)
Location is an important factor in decisions to attend annual conference	3.411	3.694	3.528	3.652
	(1.122)	(0.988)	(1.002)	(0.999)
Prefer meeting locations that are close to an airport	3.940	4.174	3.727	3.750
	(0.955)	(0.747)	(0.977)	(0.968)
Maintain membership only when attending the annual conference	3.230	3.348	3.113	2.989
	(1.250)	(1.283)	(1.260)	(1.172)

(Likert: 1 = strongly disagree, 2 = disagree, 3 = neither agree nor disagree, 4 = agree, 5 = strongly agree)

Note: Standard deviations in parentheses.

**Table 7.** Lag Selection

	Lag	LL	LR	df	p-value	FPE	AIC	HQIC	SBIC
**AEA**									
Members	4	−1211.370	117.730*	25	0	1.9e + 21*	62.6843*	64.2766*	67.0285
Registrants	1	−959.554	303.360	16	0	2.10E + 15	46.6454	46.9487*	47.4729*
**SEA**									
Members	4	−850.909	107.980*	25	0	4.1e + 16*	51.6708*	53.2825*	56.2423
Registrants	2	−784.104	47.988	16	0	2.2e + 14*	44.3299	44.8825*	45.8973
**WEAI**									
Members	4	−657.801	162.450*	25	0	4.5e + 16*	50.8534*	52.4223*	55.7576
Registrants	4	−566.712	80.408*	16	0	5.1e + 13*	42.3141*	43.3302*	45.4902

Notes:

* The test confirms the number of lags noted in the table.

**Table 8.** Johansen Tests for Cointegration

	Maximum rank	Parms	LL	Eigenvalue	Trace statistic	5% critical value
**AEA**						
Members	3	106	−1208.640	0.388	14.4531*	18.17
Registrants	1	15	−1038.980	0.487	34.1847*	34.55
**SEA**						
Members	3	106	−838.448	0.429	15.6859*	18.17
Registrants	1	31	−826.226	0.582	21.1539*	34.55
**WEAI**						
Members	3	101	−665.074	0.527	14.5460*	15.41
Registrants	2	64	−573.852	0.548	14.2792*	15.41

Notes:

* The test confirms cointegration at the rank noted in the table.

**Table 9.** VECM Estimations for Association Membership and Conference Registration

	AEA (1970–2015)		SEA (1975–2015)		WEAI (1982–2015)	
	Membership (1)	Conference Registration (2)	Membership (3)	Conference Registration (4)	Membership (5)	Conference Registration (6)
Policy 1	−2,196*	−1,894***	33.84	−101.3	203.5	−268.1*
	−1,230	−587.9	−183.5	−86.96	−212.5	−145.2
Policy 2	−353.2	2,164***				
	−974.6	−508.3				
New York	−195	1,572***				
	−421.5	−402.5				
Philadelphia	177	413				
	−829.1	−644.9				
Chicago	414.7	−181.3				
	−391.6	−419.3				
Washington D.C.	639.8	1,800***	148.9*	174.7***		
	−454.3	−476.8	−83.29	−56.33		
New Orleans			81.96	132.5**		
			−84.24	−52.4		
Los Angeles					−62.3	−154.7
					−284.2	−182.6
San Francisco					166.8*	100.3
					−96.47	−85.45
Years	YES	YES	YES	YES	YES	YES
Lags	4	1	4	2	4	4
Rank	2	1	3	1	3	2
Observations	42	45	37	39	30	30

Notes:

* significance at 10%;

** significance at 5%;

*** and significance at 1%.

Additional covariates include members, membership dues, registration fees, annual meeting registrants, PhDs, GDP (See table 6 for a listing) and their respective lags. We did not discuss or present these results because (with the exception of prices, which are discussed in another context) these variables cannot be influenced by policy.

**Table 10.** Poisson Estimation of the Number Association Memberships and Number of Meetings Attended per Year using Survey Data

	No. of Associations		No. of Meetings	
	(1)	(2)	(3)	(4)
Constant	0.5282***	0.5397***	1.0397***	1.008***
	(0.0465)	(0.0496)	(0.0528)	(0.0567)
Income	4.5499e-04	4.8150e-04	0.0284***	0.0283***
	(6.0887e-03)	(6.0853e-03)	(7.2066e-03)	(7.2129e-03)
Female	0.0258	0.0280	−0.0423**	−0.0425**
	(0.0180)	(0.0181)	(0.0212)	(0.0212)
Regional-Field Association, dummy	0.1263***	0.1255***	0.0471	0.0494
	(0.0404)	(0.0403)	(0.0456)	(0.0457)
Field Association, dummy	0.0393	0.0378	0.0243	0.0238
	(0.0267)	(0.0266)	(0.0262)	(0.0263)
Regional Association, dummy	0.1636***	0.1605***	0.0570**	0.0566**
	(0.0237)	(0.0239)	(0.0251)	(0.0251)
PhD	−0.0106	−9.8978e-03	0.0876**	0.0863**
	(0.0319)	(0.0317)	(0.0430)	(0.0429)
University	0.1672***	0.1829***	0.1301***	0.1617***
	(0.0211)	(0.0236)	(0.0257)	(0.0284)
Meetings Attended per year	0.0948***	0.0945***		
	(5.5101e-03)	(5.5070e-03)		
Membership Years	0.0339***	0.0331***	−0.0123	−0.0110
	(6.8990e-03)	(6.9050e-03)	(8.1382e-03)	(8.2993e-03)
Self-Pay	0.0303*	0.0294*	−0.1368***	−0.1353***
	(0.0167)	(0.0168)	(0.0197)	(0.0197)
Years Employed	0.0113*	4.9061e-03	−0.0343***	−0.0327***
	(6.7414e-03)	(8.1462e-03)	(7.8442e-03)	(8.9368e-03)
Employed 6+ Years		0.0814*		0.0905
		(0.0456)		(0.0568)
Employed 6+ Years × University		−0.0635		−0.1387**
		(0.0464)		(0.0573)
Observations	2,931	2,931	2,931	2,931

Notes: Robust standard errors in parentheses;

* significant at 10%;

** significant at 5%;

*** significant at 1%

## Data Availability

The association time-series data and conference data from AEA, SEA, and WEAI available upon request and subject to association approval.

## Footnotes

1 For other commentaries on women and economics, see The Economist (2017) and https://www.forbes.com/sites/johntharvey/2019/01/11/do-women-avoid-economics-or-does-economics-avoid-women/#8b303342f32c, accessed 10/06/20.

2 According to West et al. (2013), since 1990 only 13.7 percent of authorships in economics are from females.

3 Some of the larger economics meetings, like the Allied Social Science Association’s annual meeting sponsored by the American Economic Association (AEA), have begun offering childcare. However, many small and mid-size meetings still do not provide childcare, and travel costs for dependents are not typically covered by institutions.

4 Although WEAI has always focused on the Western United States as the location for its annual meeting, it has recently expanded its annual meeting venues. In addition to its domestic meeting, the association began holding biennial meetings in the Pacific Rim in 1994 and since 2015 has held its annual meeting internationally.

5 A variety of other associations were contacted for data on membership and meetings. We obtained the most comprehensive data from those presented here, representative of the three largest economics associations in the United States. We do not have enough data to include NAREA in the time series analysis.

6 All registration fees are the “standard” fees. These are the member registration fees for associations that discount registration for members and the early or (non-late) fees for those that penalize late registration.

7 To maintain confidentiality, we refer to these organizations as national, regional, field, and regional-field associations. However, we believe that each association we surveyed is representative of its respective category.

8 The number of observations in Table 3 includes all survey respondents. In Tables 4–6, we use the sample from the main empirical models in Table 10, which only include the completed surveys with full information on the variables of interest.

9 The survey instrument did not include respondent income. However, we believe that household income reflects the ability of respondents to pay for travel to conferences out-of-pocket.

10 These were identified with association data and email conversations with association leadership.

11 We identify evidence of long-run causality for both of these estimations (the error correction terms are significant).

12 Although lagged variables for members, attendance, GDP, and number of PhDs are significant in some models, the results are not displayed in Table 9, since they are not directly influenced by association policy.

13 According to the 2018 Occupational Employment Statistics, from the Bureau of Labor Statistics, Washington, DC has 6,380 economists. The next closest state is California, with 1,430 (https://www.bls.gov/oes/, accessed October 1, 2019).

14 Negative binomial models produced qualitatively similar results to the Poisson models.
